# GPG-NH_2 _acts via the metabolite αHGA to target HIV-1 Env to the ER-associated protein degradation pathway

**DOI:** 10.1186/1742-4690-7-20

**Published:** 2010-03-15

**Authors:** Alenka Jejcic, Stefan Höglund, Anders Vahlne

**Affiliations:** 1 Department of Laboratory Medicine, Division of Clinical Microbiology, Karolinska Institutet, SE-141 86 Stockholm, Sweden; 2 Department of Biochemistry, Uppsala Universitet, SE-751 23 Uppsala, Sweden

## Abstract

**Background:**

The synthetic peptide glycyl-prolyl-glycine amide (GPG-NH_2_) was previously shown to abolish the ability of HIV-1 particles to fuse with the target cells, by reducing the content of the viral envelope glycoprotein (Env) in progeny HIV-1 particles. The loss of Env was found to result from GPG-NH_2 _targeting the Env precursor protein gp160 to the ER-associated protein degradation (ERAD) pathway during its maturation. However, the anti-viral effect of GPG-NH_2 _has been shown to be mediated by its metabolite α-hydroxy-glycineamide (αHGA), which is produced in the presence of fetal bovine serum, but not human serum. In accordance, we wanted to investigate whether the targeting of gp160 to the ERAD pathway by GPG-NH_2 _was attributed to its metabolite αHGA.

**Results:**

In the presence of fetal bovine serum, GPG-NH_2_, its intermediary metabolite glycine amide (G-NH_2_), and final metabolite αHGA all induced the degradation of gp160 through the ERAD pathway. However, when fetal bovine serum was replaced with human serum only αHGA showed an effect on gp160, and this activity was further shown to be completely independent of serum. This indicated that GPG-NH_2 _acts as a pro-drug, which was supported by the observation that it had to be added earlier to the cell cultures than αHGA to induce the degradation of gp160. Furthermore, the substantial reduction of Env incorporation into HIV-1 particles that occurs during GPG-NH_2 _treatment was also achieved by treating HIV-1 infected cells with αHGA.

**Conclusions:**

The previously observed specificity of GPG-NH_2 _towards gp160 in HIV-1 infected cells, resulting in the production of Env (gp120/gp41) deficient fusion incompetent HIV-1 particles, was most probably due to the action of the GPG-NH_2 _metabolite αHGA.

## Background

The HIV-1 envelope glycoprotein (Env) is co-translationally translocated into the endoplasmic reticulum (ER) as the precursor protein gp160. It is a is a type 1 membrane protein that in the ER obtains ~30 N-linked glycans and forms 10 disulphide bonds during a slow and extensive folding process [1]. The mature gp160 trimerizes prior to its export to the Golgi, where it is being processed into the trans-membrane unit, gp41, and the highly glycosylated surface unit, gp120, which remain non-covalently associated to each other [2,3]. These trimeric gp120/gp41 complexes are then transported to the cell surface for incorporation into the assembling particles.

**Figure 1:**
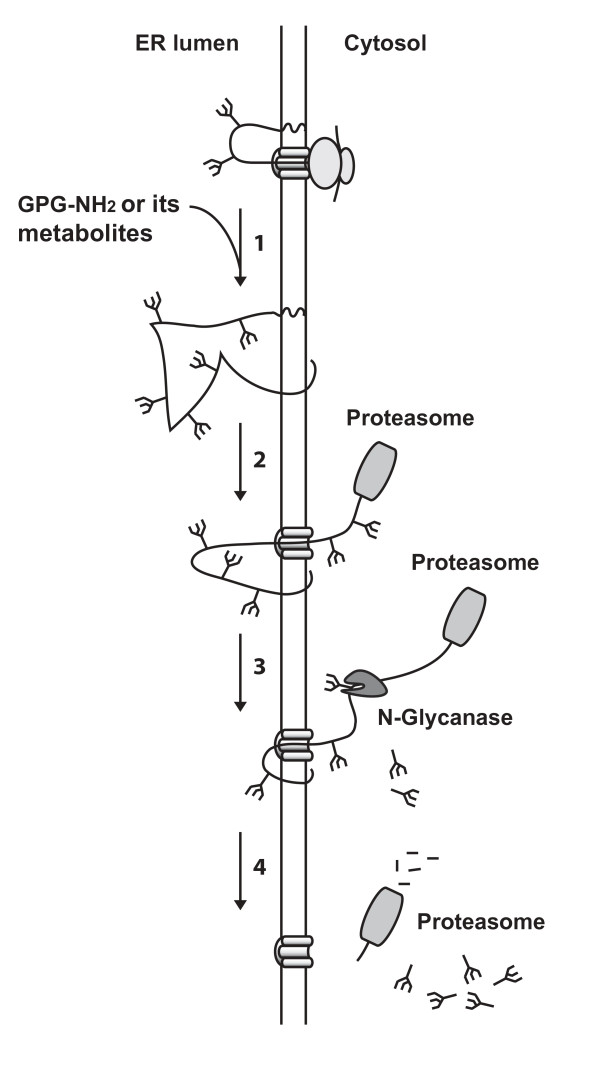
**A proposed model for how GPG-NH_2 _or its metabolites target gp160 for ERAD**. Initially, gp160 is co-translationally translocated into the ER, where its growing peptide backbone becomes glycosylated and starts to fold. (1) In the presence of GPG-NH_2 _or its metabolites gp160 folds incorrectly which targets it to ERAD. (2) Subsequently, gp160 is retro-translocated to the cytoplasm, (3) where it becomes deglycosylated by the cytosolic N-glycanase prior to (4) degradation of its peptide backbone by the proteasome.

The HIV-1 infection is initiated by its Env, where gp120 directs binding to the target cell, and gp41 mediates the fusion of the viral membrane with the host cell plasma membrane, which results in the delivery of the viral content into the cell [4].  Prevention of viral spreading by targeting viral entry can be achieved by inhibiting the function of gp120/gp41 [5,6]. However, it might also be accomplished late in the viral replication cycle by negatively affecting the maturation of gp160. This has been attempted by targeting the glycosylation of gp160 through the use of various glycosylation inhibitors, but these compounds are very non-specific and have thus far failed as therapeutic agents [7-9]. We have recently shown that the maturation of gp160 within the ER can be targeted rather specifically. Treatment of HIV-1 infected cells with the synthetic peptide glycyl-prolyl-glycine amide (GPG-NH_2_) targets gp160 to the ER-associated protein degradation (ERAD) pathway. To be initiated, this process requires the ER quality control machinery to recognize gp160 as terminally misfolded and results in its retro-translocation to the cytoplasm. In the cytoplasm the N-linked glycans are removed from the peptide chain by the N-glycanase, which gradually decreases the gp160 molecular mass prior to its degradation by the proteasome (Fig. 1) [10]. Thus, HIV-1 particles produced in the presence of GPG-NH_2 _have a significantly reduced content of gp120/gp41 on their surface [10].

**Figure 2:**
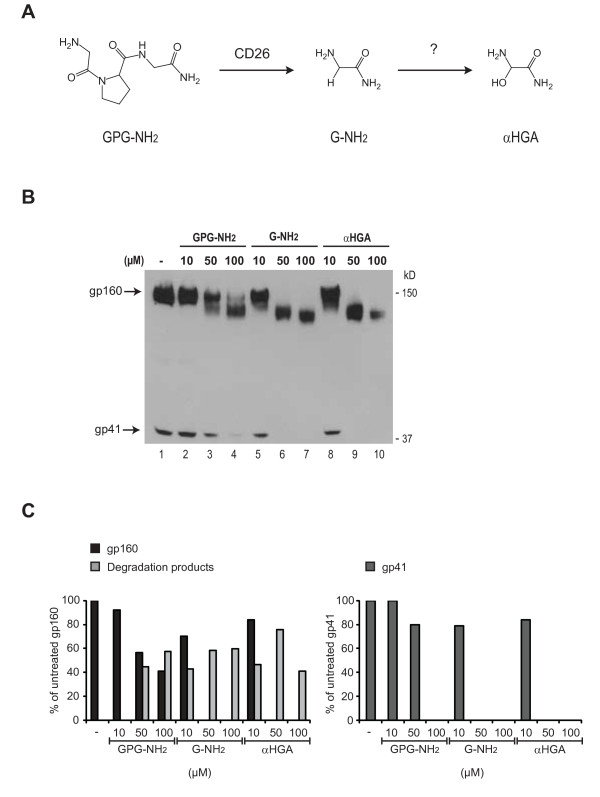
**GPG-NH_2 _and its metabolites G-NH_2 _and αHGA decrease gp160 mobility and steady-state levels**. **(A) **Scheme of GPG-NH_2 _being metabolized in cell culture medium supplemented with 10% FBS. GPG-NH_2 _is processed by CD26 (peptidyl peptidase V) to G-NH_2 _and subsequently modified into αHGA by an unidentified enzyme. **(B) **HeLa-tat III cells were transfected to express gp160. Two hours post transfection the cells were treated with the indicated concentrations of GPG-NH_2_, G-NH_2 _or αHGA and harvested 20 h post transfection. The cell lysates were separated by SDS-PAGE and immunoblotted with mAb towards gp41. **(C) **Densitometric measurement of gp160 and degradation products (left panel) and gp41 (right panel) given as percentage of total gp160 or gp41 respectively in untreated cells in (B), lane 1. The results represent the average of two experiments.

During the course of studying its anti-viral mechanism it was discovered that GPG-NH_2 _is metabolized via glycine amide (G-NH_2_) into α-hydroxy-glycine amide (αHGA) in cell culture media containing fetal bovine serum (FBS) (Fig. 2A) [11,12]. Both metabolites have been found to retain the ability to inhibit HIV-1 propagation in the presence of FBS and in serum from several other species [11]. However, in HS only αHGA still possesses its anti-viral activity against HIV-1, which indicates that the unidentified enzyme responsible for the transition of G-NH_2 _into αHGA is not present in HS [11]. This strongly suggests that the anti-viral activity previously ascribed to GPG-NH_2 _is actually an attribute of its final metabolite αHGA. In this study we therefore further examined if the potent ability of GPG-NH_2 _to target gp160 for ERAD is also dependent on it metabolizing into αHGA.

## Results

### GPG-NH_2_, G-NH_2 _and αHGA treatment all decrease the molecular mass, steady-state levels and processing of gp160

To evaluate whether the targeting of gp160 to the ERAD pathway is due to the action of GPG-NH_2_, its intermediate metabolite G-NH_2_, or its final metabolite αHGA (the structures are depicted in Fig. 2A) the respective drugs were added to HeLa-tat III cells at indicated concentrations 2 h after transfection with the gp160 expressing plasmid pNL1.5EU. Twenty hours post transfection, the cells were lysed and analyzed by immunoblotting against gp41. The mobility and steady-state levels of gp160 were affected at 50 μM and 100 μM GPG-NH_2 _(Fig. 2B, lanes 2-4). In comparison to GPG-NH_2_, both G-NH_2 _and αHGA showed a more potent activity as neither gp160 nor its processing to gp41 were detectable at 50 μM (Fig. 2B, compare lanes 6 and 9 to 3, Fig. 2C).

### αHGA does not require FBS to affect gp160

**Figure 3:**
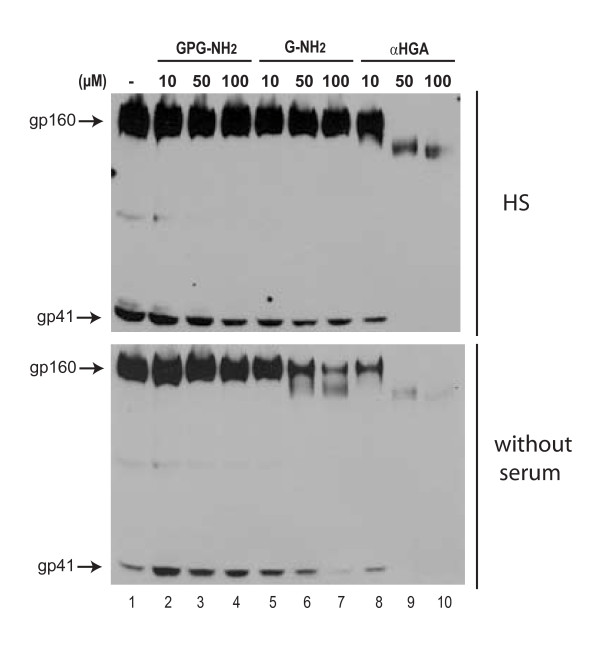
**αHGA acts on gp160 independently of supplemented serum in cell culture medium**. HeLa-tat III cells were cultured in cell culture medium supplemented with 10% FBS and transfected to express gp160 for 20 h. Two hours upon transfection the cell culture supernatants were carefully removed, the cells rinsed twice in PBS and provided with culture medium containing either 10% HS (upper panel) or no serum (lower panel) and indicated concentrations of GPG-NH_2_, G-NH_2 _or αHGA. The cell lysates were immunoblotted with mAb towards gp41.

To examine if the previously shown anti-viral activity of αHGA in HS correlates with its ability to target gp160 for ERAD, HeLa-tat III cells were transfected to express gp160 and cultured in RPMI containing HS and various concentrations of the respective drugs. As expected, GPG-NH_2 _and G-NH_2 _showed no effect, while αHGA retained its ability to target gp160 (Fig. 3, upper panel). To further test if HS is a requirement for the activity of αHGA on gp160, the transfected HeLa-tat III cells were cultured in Advanced RPMI without serum and treated with the respective drugs. Under these serum-free conditions αHGA was still able to target gp160 (Fig. 3, lower panel). Surprisingly, G-NH_2 _still had some activity towards gp160 in the absence of serum (Fig. 3, lower panel). Together these results support that the targeting of gp160 to for ERAD is dependent on the GPG-NH_2 _metabolite αHGA.

### αHGA targets gp160 for degradation more rapidly than GPG-NH_2_

**Figure 4:**
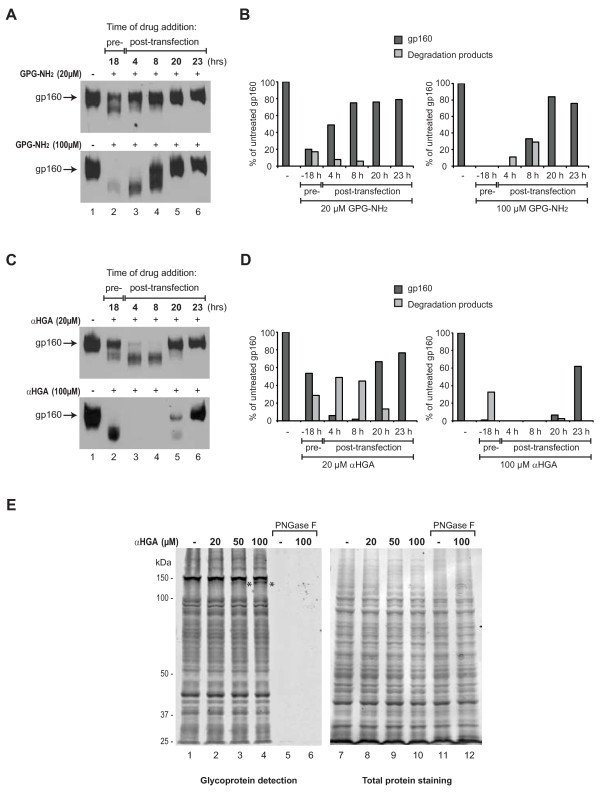
**αHGA targets gp160 for degradation more rapidly than GPG-NH_2_**. **(A) **HeLa-tat III cells were transfected to express gp160 and treated with 20 μM (upper panel) or 100 μM GPG-NH_2 _(lower panel) for the indicated times pre- or post-transfection. The cells were harvested 24 h post transfection and immunoblotted with mAb towards gp41. **(B) **Densitometric measurements of gp160 and degradation products in samples treated with 20 μM (left panel) or 100 μM GPG-NH_2 _(right panel) as described in (A) and given as percentage of total gp160 in untreated cells in (A), lane 1. **(C) **As in (A), except the cells were treated with αHGA at 20 μM (upper panel) or 100 μM (lower panel). **(D) **Densitometric measurements as described in (B) of samples treated with αHGA at 20 μM (left panel) or 100 μM (right panel) described in (C). **(E) **Glycoprotein blot of HeLa-tat III cell lysates collected from cells treated with the indicated concentrations of αHGA for 24 h and stained for total protein and subsequently probed with the lectin Concanavalin A. The asterisks highlight the decreased molecular mass species.

To investigate the temporal processing of GPG-NH_2 _to the active metabolite αHGA, the required time of cellular exposure to the respective drug for a detectable effect on gp160 was examined. HeLa-tat III cells were transfected to express gp160 and treated with 20 μM or 100 μM GPG-NH_2 _or αHGA at various time points prior to or post transfection and the cells were harvested 24 h post transfection. The strongest effect of GPG-NH_2 _on gp160, at both concentrations, was obtained when treatment was initiated 18 h prior to transfection (Fig. 4A, upper and lower panels, lane 2, and Fig. 4B). Treatment with GPG-NH_2 _starting at 4 and 8 h post transfection still significantly affected gp160 at 100 μM, but addition at 20 h and 23 h post transfection, i.e. 4 h and 1 h prior to harvesting, did not affect gp160 (Fig. 4A, lower panel, and Fig. 4B). Interestingly, the addition of 20 μM and 100 μM αHGA 18 h prior to transfection had a slightly milder effect on gp160 as compared to GPG-NH_2 _(Fig. 4C, compare lane 2 to 4A, lane 2). Thus, αHGA treatment did not benefit from early addition to the cell cultures as did GPG-NH_2_. Instead, the strongest decrease in the gp160 steady-state levels and molecular mass occurred when αHGA was added 4 or 8 h post transfection (Fig. 4C, upper and lower panels, lanes 3 and 4, Fig. 4D). Addition of αHGA, 20 h post transfection, i.e. 4 hours prior to harvest of the cells, still had an effect on gp160, while addition at 1 h prior to harvest did not (Fig. 4C upper and lower panels, lanes 5 and 6, Fig. 4D). Thus, the activity of αHGA towards gp160 requires a much shorter exposure time than that of GPG-NH_2_, supporting that GPG-NH_2 _must first be metabolized into αHGA to become active towards gp160.

We have previously shown that GPG-NH_2 _does not generally effect cellular glycoproteins, but acts rather selectively on gp160 [10]. Here, we examined the glycoprotein expression profile in the HeLa-tat III cells upon treatment with αHGA added to the cultures at seeding and collected 24 h and 48 h later. The total protein content increased two fold and three fold, respectively, during incubation time (data not shown). As for GPG-NH_2_, αHGA showed no general effect on glycoproteins at 24 h or 48 h as only a single unidentified high-molecular-mass-protein (~150 kDa) slightly increased its mobility at 50 μM and 100 μM αHGA (Fig. 4E; only 24 h blot is shown).

### αHGA decreases the content of Env in HIV-1 particles

**Figure 5:**
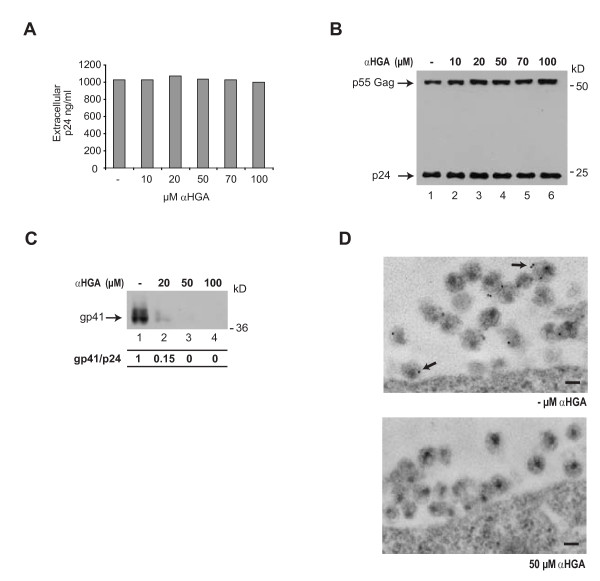
**αHGA treatment reduces HIV-1 particle content of Env**. **(A) **Chronically infected ACH-2 cells were induced with PMA for HIV-1 production and treated with the indicated concentrations of αHGA for 72 h. The viral production was determined by measuring extracellular p24 concentrations by ELISA. **(B) **Virus particles were produced as described in (A) and precipitated with polyethylene glycol followed by immunoblotting towards p24. **(C) **Immunoblot showing the amount of gp41 present in polyethylene glycol-precipitated HIV-1 particles, produced by ACH-2 as described in (A) for 48 h. The HIV-1 particle content was standardized to the extracellular p24 concentrations measured by ELISA and the gp41/p24 ratio was calculated by densitometry. **(D) **EM images of immuno-gold labeled gp41 in viral particles surrounding untreated or treated ACH-2 cells with 50 μM αHGA and induced with PMA for 72 h prior to fixation. Arrows indicate labeling of gp41 and the bars represent 100 nm.

The production of viral particles from the chronically infected ACH-2 cells, monitored by measuring the extra cellular capsid protein p24, was not affected in the presence of 10-100 μM αHGA (Fig. 5A). In addition, αHGA did not affect the viral particle content of the precursor protein p55Gag or its processing to p24 (Fig. 5B). However, treatment with αHGA resulted in a significant dose-dependent decrease in the gp120/gp41 content in the viral particles as the ratio of gp41 to p24 decreased by 85% at 20 μM αHGA to undetectable levels of gp41 at 50 μM αHGA (Fig. 5C). Also HIV-1 particles generated from ACH-2 cells in the absence or presence of 50 μM αHGA were examined for their gp120/gp41 content by immunogold labeling and transmission electron microscopy (TEM) (Fig. 5D). This further showed that αHGA decreased the incorporation of gp120/gp41 as the ratio of immuno gold labeled gp41 to the number of viral particles decreased from 0.46 (total particle number: 984) in the untreated sample to 0.07 (total particle number: 1841).

## Discussion

In this study we examined whether either of the two GPG-NH_2_-metabolites retained the ability to target gp160 for destruction in the same manner as GPG-NH_2_. Here we show that when replacing FBS with HS or in complete absence of serum the effect of GPG-NH_2 _on gp160 was completely abolished, which strongly indicates that GPG-NH_2 _is not the molecule responsible for targeting gp160 for ERAD. αHGA, on the other hand was active against gp160 both in the presence of HS and under serum free conditions. The intermediate metabolite G-NH_2 _was not able to target gp160 for destruction in HS but showed some activity in absence of serum. This means that either some of the enzymatic activity converting G-NH_2 _to αHGA remained after washing of the cells and HS prevented its conversion to αHGA or G-NH_2 _was able to affect gp160 by itself but was inhibited by HS. GPG-NH_2 _had to be added much earlier than αHGA to the cell cultures in order to be effective against gp160. The comparably slow onset of GPG-NH_2 _also supports that GPG-NH_2 _needs conversion to αHGA to target gp160 for ERAD. In addition, viral particles produced in the presence of αHGA showed a dramatic loss in their gp120/gp41 content with respect to the capsid protein p24. Therefore, the effect on gp160 resulting in reduced gp120/gp41 content in progeny viral particles rendering them fusion incompetent that was previously ascribed to GPG-NH_2 _is most likely due to its metabolite αHGA. Although, deletion of the 19 N-terminal amino acids (aa) of the 30 aa long gp160 signal sequence has been shown to render gp160 resistant to αHGA treatment, the exact site of αHGA interaction remains to be identified [10].

We have previously shown that αHGA also causes a diversity of abnormal capsid formations in progeny viral particles [11]. These two effects may be completely independent of each other as αHGA is believed to bind to the hinge region of p24 thereby preventing it from forming proper capsids [11]. However, the gp41 deficiency in the particles could also contribute to the distorted capsid formation. The exceptionally long cytosolic tail of gp41, which stretches 150 aa into the particles, interacts with p55Gag and cellular proteins and may therefore play a role in the formation of proper internal viral structures [13-16]. Although important, it is difficult to evaluate which of the two effects is mostly responsible for the overall antiviral effect and whether they are related or are two separate phenomena. In an effort to solve this, we are now trying to induce the αHGA resistant gp160 signal sequence mutations into infectious clones of HIV-1 to see if the resulting clones are infectious and if so whether αHGA retains its anti-viral activity to such mutated virus.

## Conclusions

In this study, we have reported that it is not GPG-NH_2 _but its small metabolite (90 Da) αHGA that targets gp160 for destruction via the ERAD pathway, which results in production of gp120/gp41 deficient HIV-1 progeny particles.

## Methods

### Reagents and Antibodies

GPG-NH_2 _and G-NH_2 _were purchased from Bachem Feinchemikalien and αHGA from Chemilia AB. The monoclonal antibody to gp41 (Chessie 8) [17] was obtained through the NIH AIDS Research and Reference Reagent Program, and the antibody to p24 (EF7) has previously been described [18].

### Cell Lines and Plasmids

The cell lines HeLa-tat III and ACH-2 [19,20] and the infectious HIV-1 expressing plasmid pNL4-3 [21] were obtained through NIH AIDS Research and Reference Reagent Program. The expression plasmids for gp160 from the HIV-1 strain NL43 (pNL1.5EU) [22] and for Rev (pBRev) were kindly provided by Dr. S. Schwartz (Uppsala University, Sweden). PCR^R^3.1/CAT expresses chloroamphenichol acetyltransferase and was purchased from Invitrogen.

### Transfection and drug treatments

HeLa-tat III cells (~3 × 10^5 ^cells/dish) were treated with the indicated concentrations of GPG-NH_2_, G-NH_2 _and αHGA prior to or post transfection with the gp160, and the transfection efficiency control CAT expressing plasmids using FuGENE 6 (Roche). The cells were rinsed twice in PBS and lysed 20-24 h post transfection in RIPA buffer containing 50 mM Tris-HCl pH 7.4, 1% Triton-X-100, 1% deoxycholate, 150 mM NaCl, 1 mM EDTA, 0.1% SDS and supplemented with Complete protease inhibitor cocktail (Roche).

### PNGase F digestion

Cell lysates in RIPA buffer were supplemented with 1% β-mercaptoethenol and denaturated for 10 min at 95°C. Addition of 1% NP-40 and 16 U PNGase F (New England Biolabs) was followed by incubation at 37°C for 1 h.

### Western Blot and ELISA

Cells and precipitated virus were lysed in RIPA buffer, standardized to CAT or p24 levels respectively, denatured and resolved by SDS-PAGE, transferred to nitrocellulose membranes and immunoblotted. The membranes were exposed to film for the appropriate time and band intensities were quantified using GeneTools analysis software (SynGene). For probing against cellular glycoproteins peroxidase conjugated Concanavalin A (Sigma) was used according to manufacturer's protocol. In brief, the membranes were incubated in PBS containing 2% Tween, rinsed in PBS and probed over night in solution containing 2 μg/ml Concanavalin A, 0,05%Tween, 1 mM of CaCl_2_, MnCl_2 _and MgCl_2_. For detection of total protein the membranes were stained with 0.1% Naphthol Blue Black (Sigma) dissolved in 25% isopropanol and 10% acetic acid. P24 levels in cell culture supernatants were quantified using p24-ELISA [23] and CAT concentrations in cell lysates were quantified using the CAT ELISA kit (Roche).

### Virus expression, precipitation of HIV-1 particles and immune EM

ACH-2 cells (8 × 10^5 ^cells/ml) were cultured with 100 nM 12-phorbol-13-myristate acetate (PMA) and with or without αHGA. Three days later the cell culture supernatants were collected, cleared by centrifugation at 300 × g for 10 min, passed through 0.45 μm filters and the particles were precipitated at 4°C for 48 h in 1:6 (v/v) with 40% poly ethylene glycol 6000 containing 0.667 M NaCl. The precipitated particles were allowed to sediment at 16,000 × g for 20 minutes at 4°C and the virus pellets were then dissolved in RIPA buffer.  Sample preparation of hydrated ACH-2 cells for immunocytochemical analysis was performed as previously described using 10 nm colloidal gold labeling of anti-gp41 monoclonal antibody [17,24]. Areas surrounding the infected cells were used for calculating the number of Au-labeled particles.

## Competing interests

AV is a founder and shareholder of Tripep AB and a member of its board of directors.

## Authors' contributions

AJ and AV designed the study. AJ conducted the experiments and analyzed the results. SH performed the immune TEM work and analyzed the corresponding results. AJ and AV wrote the article. All authors commented on and approved the final manuscript.
